# How to Count the Lines in Nobert’s Plates

**Published:** 1869-04

**Authors:** 


How to Count the Lines in Nobert's Plates.—The following method is given in a late number of *Silliman's Journal of Science.* "If a cobweb micrometer is used, the micrometer eye-piece should be firmly clamped in a stand screwed to the table, so that the eye-piece is close to the end of the microscope-tube, but does not touch it—a piece of black velvet being used to complete the connection. The motion of the micrometer-screw now communicates no tremor to the microscope, and all difficulty in counting the lines seen (whether real or spurious) disappears." Still better than this is the following method: The microscope being set up in a dark room, as though to take a photograph, and the eye-piece being removed, the image of the band to be counted is received on a piece of plate glass in the plateholder, and viewed with a focussing glass, on the field lens of which a black point is marked; as the focussing glass is moved on the plate from side to side, the black point is moved from line to line. The lines may thus be counted with as much ease and precision as though they were large enough to be touched with the finger.—*Monthly Microscopical Journal*, February.

